# ALDH1L1 suppresses the replication of porcine epidemic diarrhea virus by degrading viral nucleocapsid and envelope proteins

**DOI:** 10.1128/jvi.01933-25

**Published:** 2025-12-30

**Authors:** Jiarui Wang, Yan Zeng, Yuchang Liu, He Sun, Ao Gao, Dongfang Zheng, Wu Tong, Hai Yu, Hao Zheng, Guangzhi Tong, Xin Cao, Ning Kong, Tongling Shan

**Affiliations:** 1 College of Veterinary Medicine, Jilin Agricultural University 85112, Changchun, China; 2 Shanghai Veterinary Research Institute, Chinese Academy of Agricultural Sciences 118161 , Shanghai, China; 3 Jiangsu Co-Innovation Center for the Prevention and Control of Important Animal Infectious Disease and Zoonose, Yangzhou University 38043, Yangzhou, China; The University of Texas Southwestern Medical Center, Dallas, Texas, USA

**Keywords:** ALDH1L1, PEDV, N protein, E protein, STUB1, TOLLIP, autophagy

## Abstract

Porcine epidemic diarrhea virus (PEDV) is a highly pathogenic alphacoronavirus that causes severe diarrhea. It has a high fatality rate among newborn piglets, posing a considerable economic burden to the swine industry. Therefore, elucidating the host–pathogen interaction is warranted to advance precision antiviral therapies. Herein, for the first time, we noted a marked upregulation of aldehyde dehydrogenase 1 family member L1 (ALDH1L1) during PEDV infection. Furthermore, ALDH1L1 exerts its antiviral effects by specifically binding to the viral nucleocapsid (N) and envelope (E) proteins and mediating their degradation via the autophagosome–lysosomal degradation pathway. Additional experiments revealed that this degradation process is mediated via the interactions of ALDH1L1 with the E3 ubiquitin ligase STUB1 and the cargo receptor TOLLIP, eliminating the N and E structural glycoproteins via the autophagolysosomal pathway. Our study findings suggest the ALDH1L1-STUB1-TOLLIP axis as a novel antiviral target and propose a new strategy for viral clearance based on the degradation of host protein. Furthermore, our research provides valuable information on how host antiviral factors impede PEDV replication as a regulator of the protein degradation pathway.

**IMPORTANCE:**

Porcine epidemic diarrhea virus (PEDV) is a highly pathogenic alphacoronavirus that causes fatal hemorrhagic gastroenteritis among neonatal piglets. This causes significant financial losses. During infection, certain host factors can activate the innate immune regulatory network to antagonize the viral replication cycle, interfere with the virus invasion, inhibit virus replication, prevent virus assembly and release, and enhance the host’s immune response. Our study revealed that the host metabolic enzyme ALDH1L1 acts as a novel antiviral restriction factor that mediates the autophagy–lysosome–targeted degradation of viral structural proteins (N/E) via the STUB1 (E3 ubiquitin ligase)–TOLLIP (autophagy adaptor protein) axis. Our study findings offer new perspectives on the mechanism by which host antiviral factors inhibit PEDV by regulating the protein degradation pathway.

## INTRODUCTION

Porcine epidemic diarrhea virus (PEDV) is the causative agent of porcine epidemic diarrhea (PED), which manifests as acute diarrhea, vomiting, and dehydration in clinical settings. It has a high mortality rate (up to 100% in neonatal piglets) (1, 2). In 1971, PEDV was initially identified in the United Kingdom (3), causing serious health challenges and considerable economic losses to the swine industry globally. PEDV is a highly pathogenic enterovirus, necessitating urgent prevention and control measures. Transmission primarily occurs via the fecal–oral and nasal inhalation routes, posing significant health risks and substantial economic burdens to the swine industry globally (4). The predicted efficacy of existing vaccines has been dramatically undermined owing to the discovery of PEDV variants in 2010 (5–7). Although active vaccination efforts have been taken, the infection and mortality rates of PEDV remain unacceptably high (8, 9). Subsequently, innovative antiviral strategies are urgently warranted to effectively prevent PEDV infection. PEDV belongs to the genus *Alphacoronavirus* and the order Nidovirales (10). It possesses a 28 kb long positive-sense, single-stranded RNA genome. The genome contains four structural glycoproteins: membrane (M), spike (S), nucleocapsid (N), and envelope (E). In addition, it contains 16 nonstructural proteins (nsp1–nsp16), with a minimum of seven open reading frames (11–13). The M protein is responsible for determining virus morphology and regulating host signaling pathways; the S protein mediates virus invasion and immunological response; the E protein mediates virus assembly, release, and virulence; and the N protein protects genomic RNA and participates in virus replication and immunity.

ALDH1L1 belongs to the aldehyde dehydrogenase protein family, also called 10-formyl tetrahydrofolate dehydrogenase. This gene is primarily localized in the cytoplasmic matrix. It functions as a folate-metabolizing enzyme, with characteristics similar to tumor suppressors. It plays a role in regulating folate metabolism and DNA/RNA biosynthesis (14–18). Research suggests that ALDH1L1 is intricately associated with the inflammatory process and may affect the inflammatory response as a mitochondrial ROS modulator, a mitochondrial respiratory function regulator, and an NLRP3/IL-1β pathway mediator (19). ALDH1L1 expression is significantly downregulated across diverse cancer types. This implies that the absence of this folate-metabolizing enzyme may provide a selective advantage to rapidly growing cancer cells (19, 20). However, when its overexpression is recovered, it exhibits considerable antiproliferative effects by depleting intracellular 10-formyl tetrahydrofolate and affecting cancer cell migration and invasion via specific folate-dependent mechanisms involving invasive phenotypes (21). At present, studies on ALDH1L1 function have primarily focused on tumor metabolic regulation, and systematic studies on ALDH1L1 in viral infection remain limited (19). The potential roles of ALDH1L1 in viral replication, the host immune response, or pathological processes remain unelucidated.

Autophagy is a ubiquitous and highly conserved process of intracellular degradation via lysosomes (22, 23). Both internal and external stress signals can trigger this process, removing proteins, organelles, pathogens, or specific aggregates from the body ((24–26). Based on the cargo receptor involved, autophagy is categorized into two types: selective and nonselective (27). Selective autophagy not only plays a central role in maintaining the structural integrity and homeostasis of intracellular organelles but also acts as a vital defender in the host’s inherent immune response against pathogen infection (28–30). Since the COVID-19 pandemic, several studies have revealed that coronaviruses extensively interact with autophagy in cells (31). Previous literature suggests that autophagy is significantly associated with PEDV progression. For example, bone marrow stromal cell antigen 2 can be induced by IRF1 to diminish PEDV replication by using selective autophagy to target and degrade the viral N protein (32). POLM inhibits PEDV proliferation by recruiting E3 ubiquitin ligase MARCH8 to catalyze PEDV structural protein (N, S2, and M) ubiquitination and by directing cargo receptor p62 to autophagolysosomes for degradation (33). Various host proteins, including hnRNP K, PRPF19, RALY, and BTG3, play a role in ubiquitinating and breaking down PEDV structural proteins by regulating the MARCH8-NDP52-mediated selective autophagy pathway (34–37).

In this study, we elucidated a new antiviral mechanism by which ALDH1L1 mediates the breakdown of viral structural proteins via a selective autophagy pathway. This mechanism suggests that ALDH1L1 first recruits STUB1 to catalyze the ubiquitination of viral N and E proteins. Then, TOLLIP precisely identifies the ubiquitinated proteins. Finally, viral proteins are cleared, and the PEDV replication cycle is effectively inhibited via the autolysosome pathway. Therefore, our study findings demonstrate the potential of ALDH1L1 as a viable therapeutic target for managing PED.

## RESULTS

### ALDH1L1 expression increases after PEDV infection

**Fig 1:**
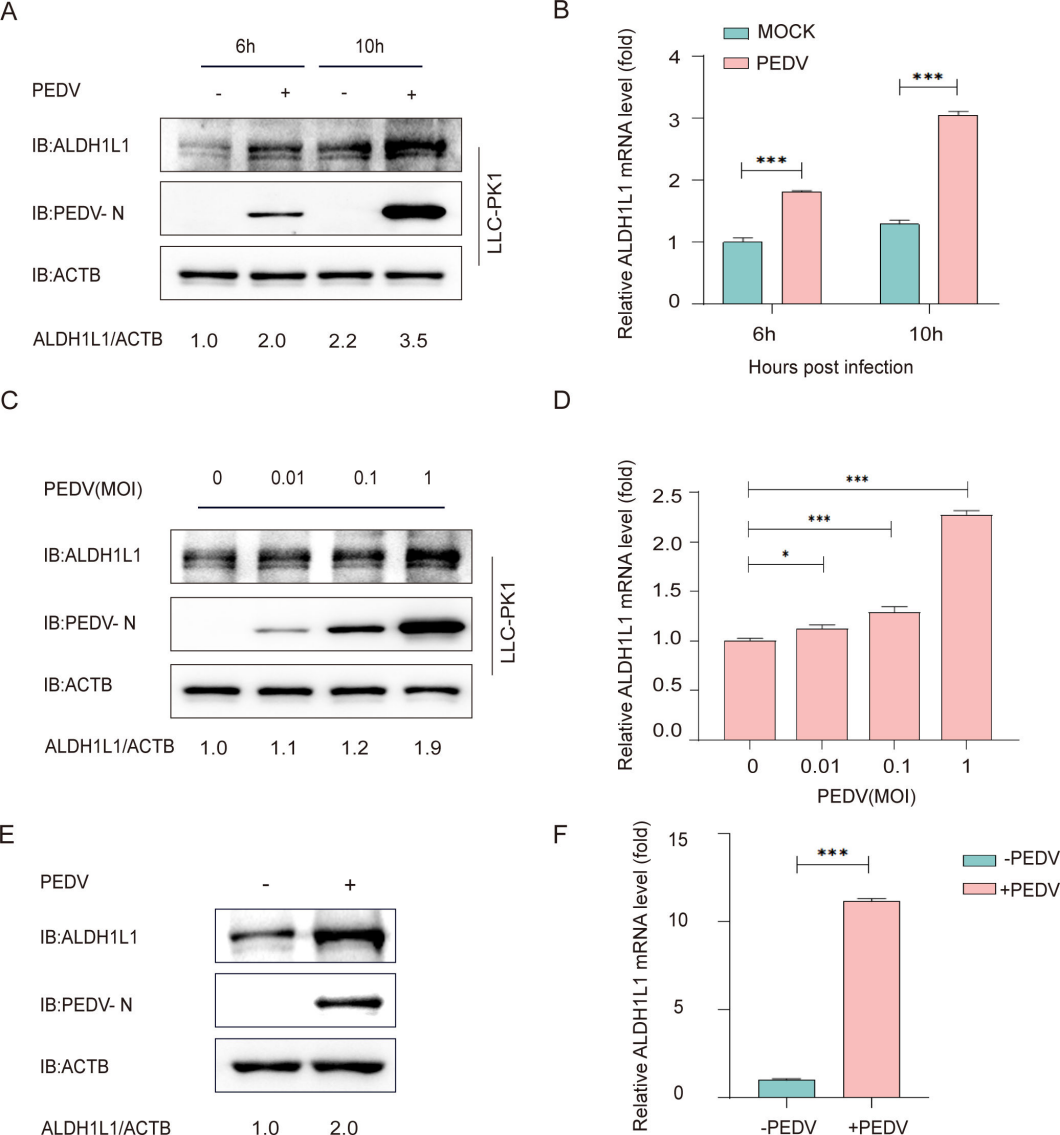
PEDV infection upregulates ALDH1L1 in LLC-PK1 cells. (**A and B**) Analyses of the mRNA and protein expression of ALDH1L1 in PEDV-challenged (MOI = 0.1) LLC-PK1 cells using western blotting and RT-qPCR. (**C and D**) LLC-PK1 cells were collected at 10 h after challenge with various MOIs of PEDV. The protein and mRNA expression of ALDH1L1 was assessed via western blotting and RT-qPCR. (**E and F**) Collect the small intestinal tissues of pigs in the PEDV-infected group and the control group without infection. After grinding, the proteins and mRNA were extracted and analyzed by western blotting and qRT-PCR. The data are expressed as means ± standard deviations from triplicate individual experiments. *P < 0.05, **P < 0.01, and ***P < 0.001 (two-sided Student’s *t*-test).

Studies on the interactions of PEDV structural glycoproteins with the host to regulate their replication are becoming increasingly comprehensive. We used mass spectrometry and identified potential host proteins that interact with the PEDV structural proteins. Among the identified proteins, ALDH1L1 captured our interest. Therefore, it was selected for further investigation. To determine the effect of ALDH1L1 expression on PEDV infection, we collected and examined LLC-PK1 cells after challenge with PEDV (JS-2013 strain). The multiplicity of infection (MOI) was 1, as in a previous study (32). Western blotting and reverse transcription-quantitative PCR (RT‒qPCR) (Table 1) revealed increased ALDH1L1 expression in cells after PEDV infection compared with uninfected cells (Fig. 1A). Consistent with its protein levels, the mRNA expression of ALDH1L1 was also higher in PEDV-challenged cells than in uninfected cells (Fig. 1B). To confirm these results, PEDV was inoculated into the LLC-PK1 cells at an MOI of 0, 0.01, 0.1, and 1, respectively. Western blotting and RT‒qPCR results demonstrated that the protein and RNA levels of ALDH1L1 gradually increased with increasing viral presence (Fig. 1C and D). Furthermore, we used small intestinal tissues of pigs uninfected or infected with PEDV to analyze ALDH1L1 expression. Western blotting and RT-qPCR assays showed that the protein and mRNA levels of ALDH1L1 in porcine small intestinal tissue cells from the PEDV-infected group were significantly higher than those in the non-infected control group (Fig. 1E and F), indicating that the endogenous ALDH1L1 would be upregulated during PEDV infection. Collectively, these findings indicate that the PEDV challenge upregulates endogenous ALDH1L1 in host cells.

**TABLE 1:** Sequences of primers and siRNAs applied in this study

Purpose	Names	Sequences (5′ −3′)
Real-time PCR	PEDV *N* forward PEDV *N* reverse	GAGGGTGTTTTCTGGGTTG CGTGAAGTAGGAGGTGTGTTAG
	*pALDH1L1* forward	CATGTCGCCGTCAGCAAAT
	*pALDH1L1* reverse	AGATCGGCAACCCTCTCGA
	*ACTB* forward	TCCCTGGAGAAGAGCTACGA
	*ACTB* reverse	AGCACTGTGTTGGCGTACAG
	*pGAPDH* forward	ATGGATGACGATATTGCTGCGCTC
	*pGAPDH* reverse	TTCTCACGGTTGGCTTTGG
siRNA		
	*si-ALDH1L1* sense	CCCUGAGCAAUGUGAAGAATT
	*si-ALDH1L1* antisense	UUCUUCACAUUGCUCAGGGTT
	*si-Tollip* sense	GACUCUUUCUAUCUCGAGATT
	*si-Tollip* antisense	UCUCGAGAUAGAAAGAGUCTT
	*si-STUB1* sense	GUGGCAAGAUCAGCUUUGATT
	*si-STUB1* antisense	UCAAAGCUGAUCUUGCCACTT
	NC sense	UUCUCCGAACGUGUCACGUTT
	NC antisense	ACGUGACACGUUCGGAGAATT

### ALDH1L1 suppresses PEDV replication

**Fig 2:**
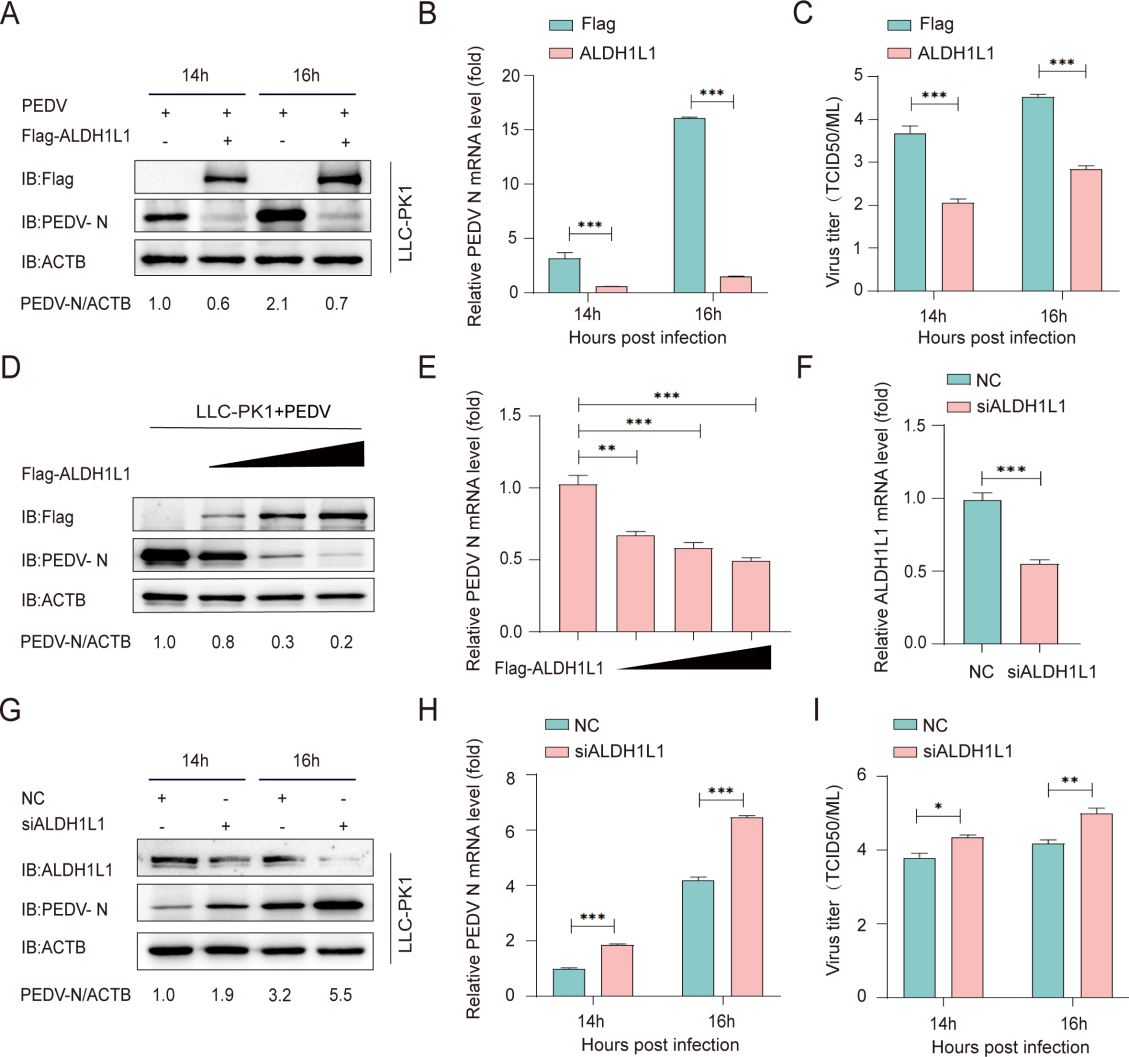
ALDH1L1 suppresses PEDV replication in cells. (**A–C**) LLC-PK1 cells were challenged with PEDV (MOI = 0.01) after transfection with ALDH1L plasmids. Virus replication was analyzed via western blotting, RT-qPCR, and TCID_50_ results. (**D and E**) After transfection with an enhancing Flag-ALDH1L1 plasmid, LLC-PK1 cells were challenged with PEDV (MOI = 0.01). The cellular samples and supernatants were collected to analyze PEDV replication via western blotting and RT-qPCR. (**F**) After transfecting LLC-PK1 cells with ALDH1L1-targeting siRNAs, the effectiveness of siRNA interference was detected via RT-qPCR. (**G–I**) After PEDV challenge (MOI = 0.01), LLC-PK1 cells were transfected with ALDH1L1 or negative control siRNA. Viral replication was investigated via western blotting, RT-qPCR, and TCID_50_ results. The data are expressed as means ± standard deviations from triplicate samples. *, *P* < 0.05; **, *P* < 0.01; ***, *P* < 0.001 (two-sided Student’s *t*-test).

The increased expression of ALDH1L1, owing to PEDV infection, suggests its underlying implication in PEDV proliferation. To elucidate ALDH1L1 function in PEDV infection, we initially investigated whether ALDH1L1 affects PEDV replication *in vitro*. For this, LLC-PK1 cells were transfected with Flag-ALDH1L1, followed by infection with PEDV (MOI = 0.01) at 24 h after transfection. The infected culture supernatants alongside cells were collected at the indicated time points. PEDV replication was measured via western blotting, RT-qPCR, and median tissue culture infective dose (TCID50) determination. We noted substantial reductions in the mRNA and protein expression of the viral N protein in ALDH1L1-translated cells compared with control cells (Fig. 2A through C). Consistent with these findings, a gradient overexpression of ALDH1L1 in LLC-PK1 cells led to a gradient inhibition of virus proliferation (Fig. 2D and E). Collectively, these results suggest the role of ALDH1L1 in inhibiting PEDV replication. To validate the mediatory function of ALDH1L1 in PEDV replication, LLC-PK1 cells were transfected with ALDH1L1 siRNA for 24 h and subsequently infected with PEDV (MOI = 0.1) (Table 1). Western blotting, RT-qPCR, and TCID50 results revealed that the intracellular silencing of ALDH1L1 enhanced PEDV replication (Fig. 2F through I). Therefore, these results confirm that ALDH1L1 can significantly inhibit PEDV replication.

### ALDH1L1 interacts with and degrades PEDV N and E proteins via the selective autophagy pathway

**Fig 3:**
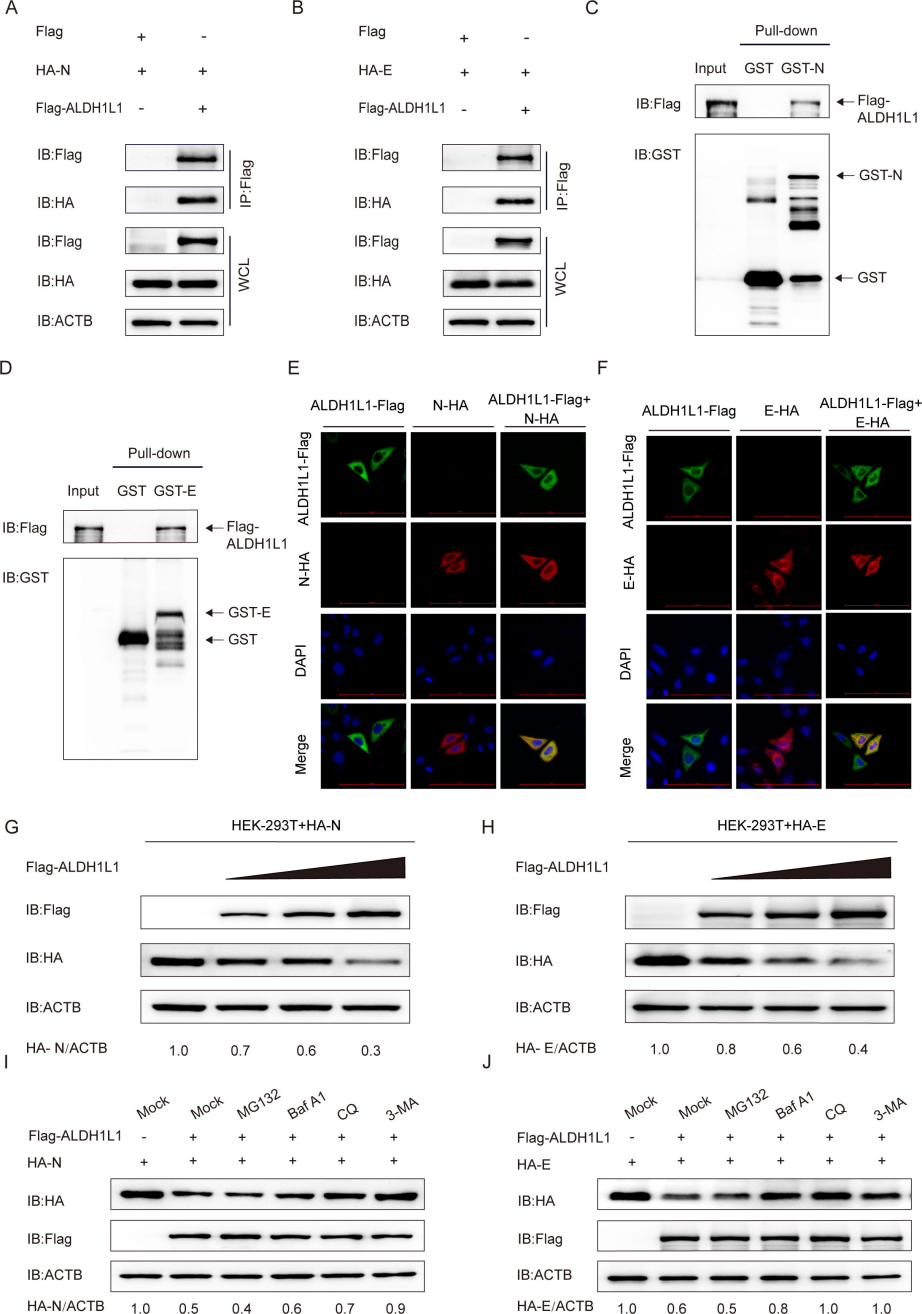
ALDH1L1 interacts with and degrades PEDV N and E proteins via the selective autophagy pathway. (**A and B**) HEK 293T cells were transfected with plasmids coding HA-N or HA-E and Flag-ALDH1L1. Co-IP assay was performed using anti-Flag-bound beads. (**C and D**) After inducing Flag-ALDH1L1 and GST-N or GST-E expression in the BL21(DE3) bacterial strain, the associations of ALDH1L1 with N or E proteins were analyzed via the GST pull-down assay. (**E and F**) After transfection with HA-N or HA-E and Flag-ALDH1L1, HeLa cells were labeled with antibodies and nuclear DAPI for confocal immunofluorescence microscopy. Scale bars = 100 µm. (**G and H**) After transfecting HEK 293T cells with varying concentrations of Flag-ALDH1L1, HA-N, or HA-E, western blotting was performed to assess N and E protein expression. (**I and J**) HEK 293T cells were transfected with plasmids coding the Flag-ALDH1L1 plasmid and HA-N or HA-E, followed by treatment with BafA1 (50 µM), CQ (50 µM), MG132 (5 µM), or 3-MA (1 mM) for 10 h. The resulting cellular lysates were subjected to western blotting.

To demonstrate whether the structural proteins of PEDV and ALDH1L1 interact to impede virus proliferation, we subjected HEK 293T cells that were cotransfected with plasmids encoding PEDV M, S1, S2, E, and N proteins, as well as ALDH1L1 protein, to a coimmunoprecipitation (co-IP) assay. We noted efficient co-IP of ALDH1L1 with the N and E proteins (Fig. 3A and B). Furthermore, the GST pull-down assay revealed that ALDH1L1 directly bound to the PEDV N and E proteins (Fig. 3C and D). Next, to further elucidate the localization of PEDV N/E and ALDH1L1, we cotransfected HeLa cells with plasmids coding Flag-ALDH1L1 and N/E-HA. Their localization was observed under a confocal microscope. As illustrated in Fig. 3E and F, the N/E proteins colocalized with ALDH1L1 in the cytoplasm.

During the coexpression of ALDH1L1 and N/E proteins in HEK 293T cells, we observed that PEDV N/E protein abundances decreased with increasing ALDH1L1 protein expression (Fig. 3G and H). In eukaryotic cells, the ubiquitin–proteasome and autophagy–lysosome pathways are the two core degradation mechanisms that regulate intracellular protein homeostasis (37). To verify the degradation pathway predominantly responsible for ALDH1L1-mediated breakdown of PEDV N/E proteins, we transfected Flag-ALDH1L1 and HA-N/E plasmids into HEK 293T cells supplemented with the protease inhibitor MG132 (5 µM), alongside autophagy inhibitors, such as bafilomycin A1 (BafA1; 50 µM), 3-methyladenine (3-MA; 1 mM), and chloroquine (CQ; 50 µM), for 10 h. Western blotting revealed that treatment with BafA1, CQ, and 3-MA inhibited ALDH1L1-mediated degradation of N and E proteins. In contrast, MG132 did not exhibit this effect, suggesting that ALDH1L1 degraded PEDV N and E proteins via the autophagy–lysosome pathway (Fig. 3I and J). Collectively, these findings suggest that ALDH1L1 promotes the autophagic breakdown of the PEDV N and E proteins.

### ALDH1L1 interacts with STUB1 to ubiquitinate N and E proteins

**Fig 4:**
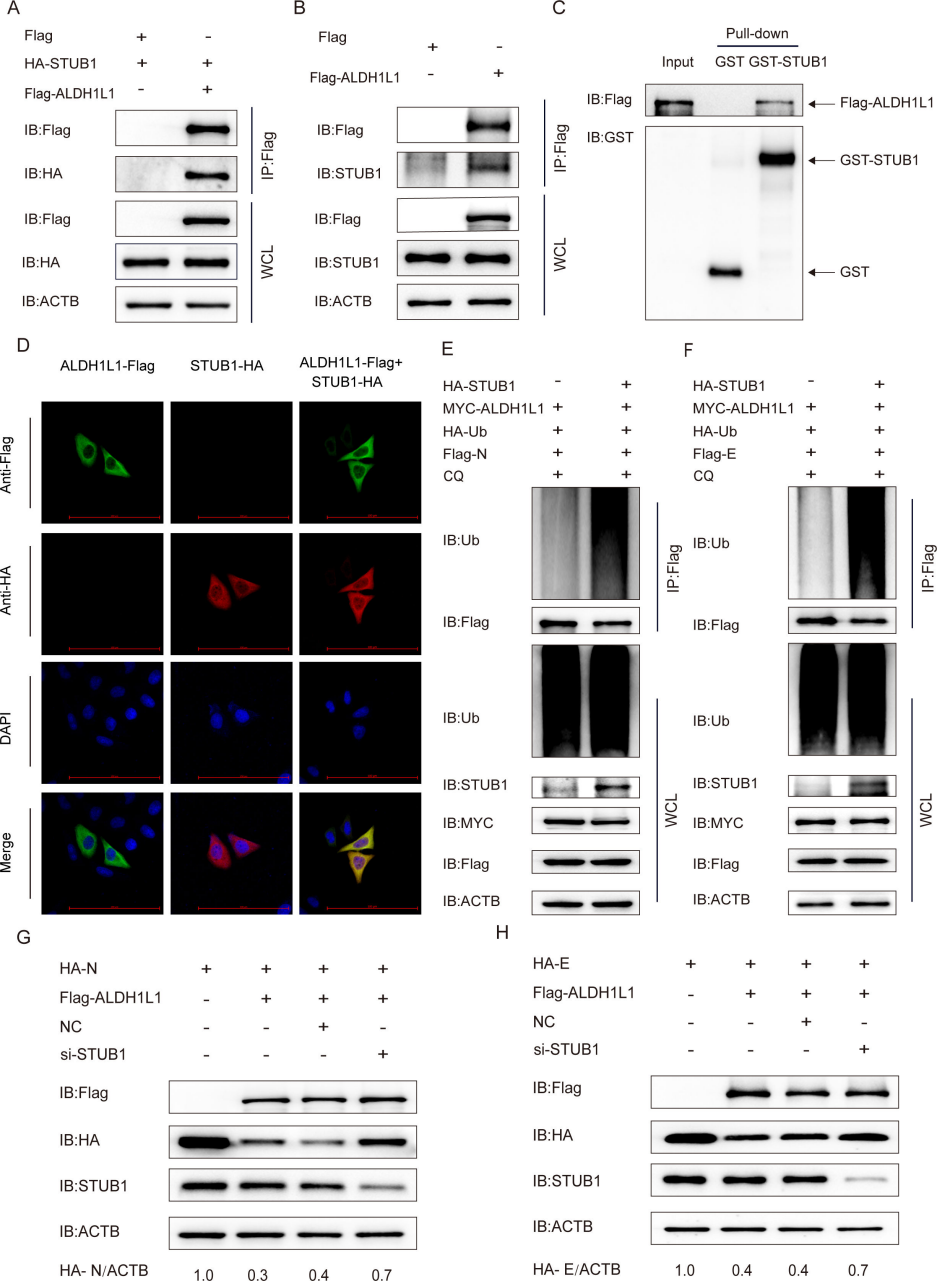
ALDH1L1 inhibits PEDV replication via the E3 ubiquitin ligase STUB1. (**A**) After HEK 293T cells were cotransfected with plasmids encoding HA-STUB1 and Flag-ALDH1L1, the Co-IP assay was performed using anti-Flag-bound beads. (**B**) After HEK 293T cells were transfected with plasmids coding Flag-ALDH1L1, Co-IP assays were performed using an anti-Flag antibody. (**C**) After inducing GST-STUB1 and ALDH1L1 expression in the BL21(DE3) strain, the interaction between ALDH1L1 and STUB1 was examined via the GST pull-down assay. (**D**) After cotransfecting HeLa cells with Flag-ALDH1L1 and STUB1-HA for 24 h via incubation with an anti-Flag Mab, ALDH1L1 and STUB1 colocalization was confirmed via confocal immunofluorescence microscopy. Scale bars = 100 µm. (**E and F**) After cotransfection with Myc-ALDH1L1, HA-Ub, HA-STUB1, and Flag-N, or Flag-E, HEK 293T cells were treated with CQ and collected. The ubiquitinated N and E proteins were subjected to western blotting after immunoprecipitation with an anti-Flag antibody. (**G and H**) After cotransfecting HEK 293T cells with siRNAs (negative control or STUBA siRNA) and HA-N or HA-E and Flag-ALDH1L1-coding plasmids, western blotting was performed with an anti-HA antibody.

During selective autophagy, viral proteins can form molecular tags through E3 ubiquitin ligase-mediated ubiquitination. In turn, cargo receptors (e.g., OPTN and p62/SQSTM1) recognize and anchor to the autophagosome membrane. Finally, lysosomal hydrolases achieve the targeted degradation of viral proteins via the acidic microenvironment formed by the fusion of autophagosomes and lysosomes (38). In the present study, we revealed the selective autophagic degradation of PEDV N and E proteins by ALDH1L1. To screen the ubiquitin ligases that interact with ALDH1L1, the interaction between ALDH1L1 and various ubiquitin ligases was analyzed via the Co-IP assay. We noted that ALDH1L1 bound to the E3 ubiquitin ligase STUB1 *in vitro*. This interaction was further confirmed via co-IP with the endogenous STUB1 protein by ALDH1L1 in HEK 293T cells (Fig. 4A and B). Moreover, the GST pull-down and confocal immunofluorescence assays revealed that ALDHIL11 and STUB1 directly bound in the cytoplasm, exhibiting colocalization (Fig. 4C and D). To determine the role of STUB1 in ALDH1L1-elicited autophagic breakdown of N and E proteins, we overexpressed STUB1 in HEK 293T cells. We noticed that STUB1 overexpression significantly enhanced the ubiquitination of N and E proteins (Fig. 4E and F). These findings suggest the function of STUB1 in enhancing the ubiquitination of N and E proteins during ALDH1L1-mediated autophagic breakdown. Next, we transfected STUB1 siRNA into HEK 293T cells to verify the importance of STUB1 in ALDH1L1-induced PEDV N and E protein degradation. Western blotting revealed that interference with STUB1 inhibited the ALDH1L1-triggered degradation of the viral N and E proteins (Fig. 4G and H). Overall, these findings suggest that STUB1 is an important E3 ubiquitin enzyme that participates in the PEDV structural protein breakdown by ALDH1L1.

### TOLLIP assists ALDH1L1-mediated PEDV N and E protein degradation

**Fig 5:**
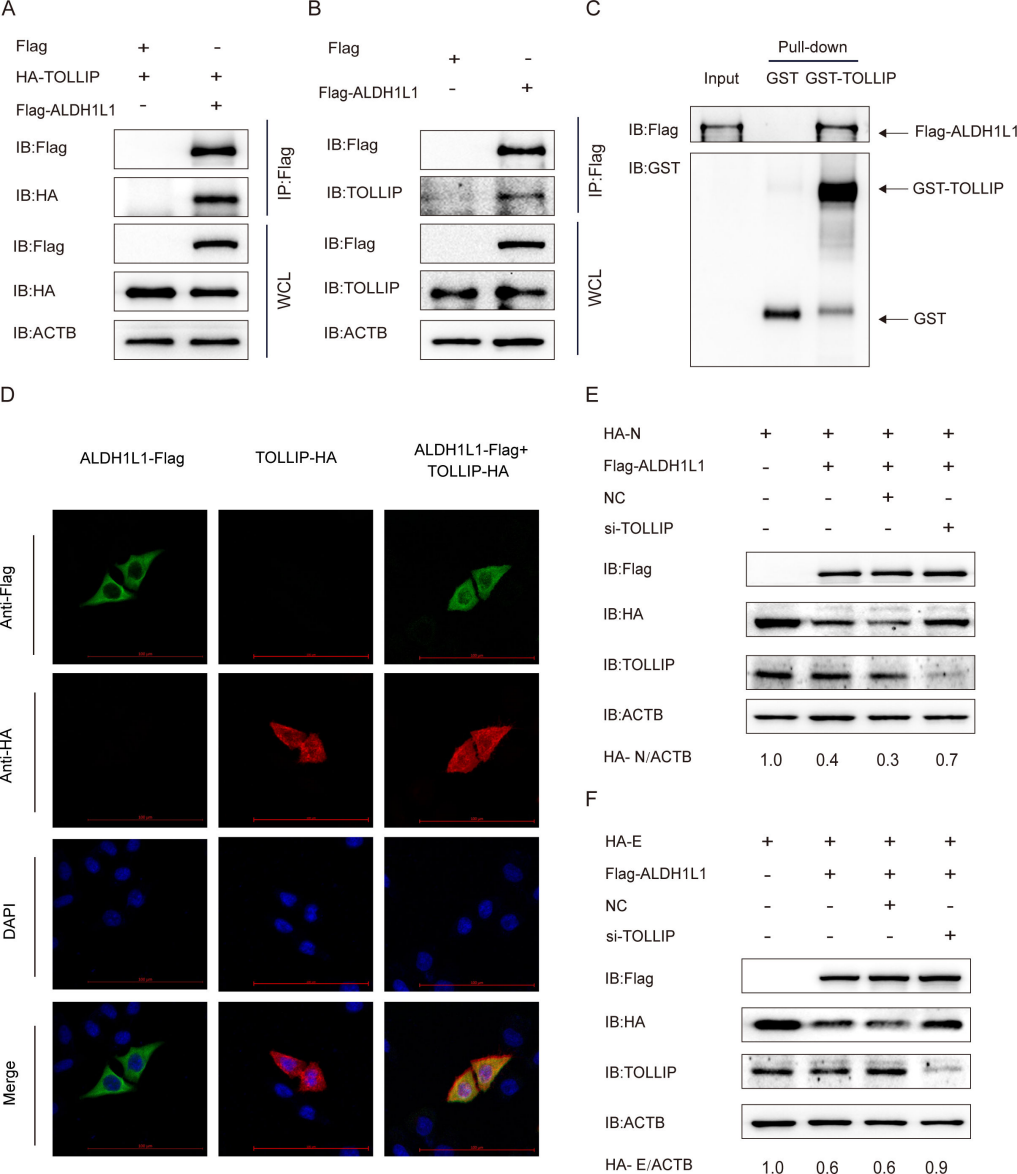
ALDH1L1 suppresses PEDV replication via the cargo receptor TOLLIP. (**A**) After cotransfecting HEK 293T cells with plasmids coding HA-TOLLIP and Flag-ALDH1L1, a Co-IP assay was performed with anti-Flag-bound beads. (**B**) After transfecting HEK 293T cells with plasmids encoding Flag-ALDH1L1, Co-IP assays were performed using an anti-Flag antibody. (**C**) After inducing GST-TOLLIP and ALDH1L1 expression in the BL21(DE3) strain, the interaction between ALDH1L1 and TOLLIP was examined via the GST pull-down assay. (**D**) HeLa cells were transfected with plasmids coding Flag-ALDH1L1 and HA-TOLLIP. Then, the cells were incubated with specific antibodies and visualized under a confocal immunofluorescence microscope. Scale: 100 µm. (**E and F**) After cotransfecting HEK 293T cells with siRNAs (negative control or TOLLIP siRNA) and HA-N or HA-E and Flag-ALDH1L1-coding plasmids, western blotting was performed using an anti-HA antibody.

During virus infection, cargo receptors act as a mediator for the selective degradation of virus particles or proteins by transporting substrates to autophagosomes, thereby inhibiting virus proliferation (39). To screen the cargo receptor used in ALDH1L1-mediated protein degradation, we analyzed the interaction between ALDH1L1 and multiple cargo receptors (NDP52, TOLLIP, and OPTN). We noted that the cargo receptor TOLLIP interacts with ALDH1L1. This was confirmed via the GST pull-down and Co-IP assays (Fig. 5A through C). Furthermore, the cytoplasmic colocalization of TOLLIP and ALDH1L1 was confirmed using the immunofluorescence confocal assay (Fig. 5D). We hypothesized that TOLLIP can help ALDH1L1 in transporting ubiquitylated viral structural proteins to autolysosomes for degradation. To explore this hypothesis, we used ALDH1L1 siRNA to inhibit TOLLIP expression in HEK 293T cells (Table 1). Flag-ALDH1L1, HA-N, or HA-E-coding plasmids were simultaneously transfected into the cells. As expected, decreased TOLLIP expression inhibited ALDH1L1-triggered degradation of PEDV N and E proteins (Fig. 5E and F). Collectively, these results suggest that TOLLIP helps ALDH1L1 in the selective autophagic degradation of viral structural proteins.

## DISCUSSION

**Fig 6:**
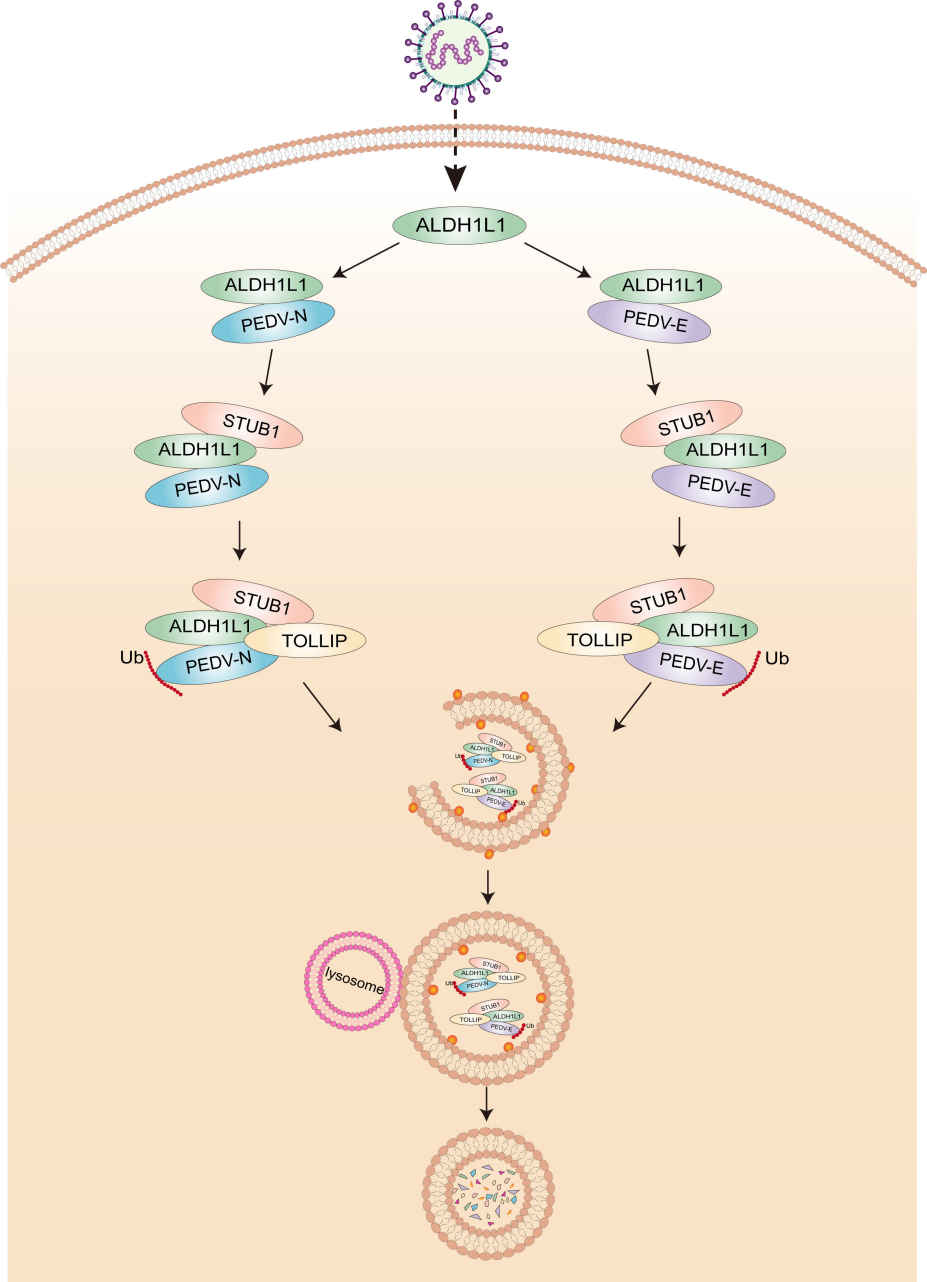
Antiviral mechanism by which ALDH1L1 inhibits PEDV replication. During infection with PEDV, ALDH1L1 interacts with the E3 ubiquitin ligase STUB1 to ubiquitinate the PEDV N or E protein. Subsequently, it recruits the cargo receptor TOLLIP to recognize and autophagosomally transport ubiquitinated N or E protein for degradation.

The global PEDV outbreak has placed a considerable financial burden on the worldwide swine farming industry (9). However, the implementation of effective PEDV prevention and control strategies remains a significant challenge, primarily owing to the rapid genetic diversification driven by the high frequency of RNA virus mutations, complicating the management of viral threats (5, 40, 41). Therefore, understanding the molecular interactions between PEDV and host antiviral factors is essential not only for discovering key pathways involved in viral escape from host innate immunity but also for establishing a theoretical foundation for developing innovative vaccines and targeted drugs aimed at blocking viral mechanisms. Our study is the first to identify the novel function of ALDH1L1 in combating PEDV infection. Our study findings suggest that ALDH1L1 can inhibit PEDV proliferation by degrading the viral N and E proteins via the ALDH1L1-STUB1-TOLLIP autophagy pathway (Fig. 6).

The ALDH1L1 protein comprises four homologous subunits, each containing 902 amino acid residues, forming a stable tetrameric structure (42). Each subunit comprises three functionally distinct domains: an N-terminal catalytic domain with a conserved aldehyde dehydrogenase active center, an oligomerization domain that mediates intersubunit interactions, and a regulatory domain that participates in cofactor binding. ALDH1L1 exhibits predominant hepatic expression, playing essential roles in one-carbon metabolism. This encompasses *de novo* nucleotide biosynthesis, NAD(P)H production, and cellular methylation (20, 43, 44). Research has revealed that ALDH1L1 expression is significantly downregulated in hepatocellular carcinoma (HCC) tissues. This suggests its role as a viable prognostic biomarker for the survival of individuals with HCC (45). Nevertheless, the molecular regulatory mechanisms of ALDH1L1 in virus infection models remain unclear. Our findings revealed the significant upregulation of ALDH1L1 expression in PEDV-challenged cells. Furthermore, ALDH1L1 overexpression notably inhibited PEDV replication in LLC-PK1 cells across various infection stages. In addition, we noted interactions between ALDH1L1 and the viral N and E proteins. These findings suggest that ALDH1L1 can act as a therapeutic target for modulating PEDV infection. We can utilize the molecular characteristics of ALDH1L1 to design drugs that specifically activate ALDH1L1, thereby enhancing its antiviral activity and inhibiting viral replication.

As an evolutionarily conserved lysosome-mediated catabolic pathway, autophagy plays an essential role in antiviral immunological responses and initiates pattern recognition receptor-mediated innate immune signaling cascades (46, 47). Importantly, selective autophagy specifically engages with autophagic cargo receptors (e.g., NDP52 and T6BP) via the ubiquitination signal decoding system to achieve the targeted clearance of invading pathogens, thereby maintaining intracellular homeostasis (48). For example, the measles virus C protein/V protein complex and nucleocapsid proteins can specifically bind to NDP52 and T6BP, respectively. These interactions promote the degradation of viral proteins and the effective inhibition of the viral life cycle by activating the autophagy–lysosomal degradation pathway (49, 50). Hepatitis C virus nonstructural protein NS5A can be targeted by the endoplasmic reticulum transmembrane protein SCOTIN for autophagosomal degradation (51). In the present study, we observed interactions between the cargo receptor TOLLIP and PEDV N and E glycoproteins, thereby participating in ALDH1L1-mediated virus protein degradation.

STUB1, an E3 ubiquitin ligase typified by a ring finger-like U-box domain, plays an essential role in antiviral innate immunological response via the ubiquitin–proteasome pathway (52–54). For example, during the inhibition of vesicular stomatitis and bovine ephemeral fever virus infections, STUB1 participates in the RACK1-mediated ubiquitination and degradation of MAVS (55). Previous studies have revealed that the host protein PGAM5 inhibits PDCOV by interacting with the transporter receptor P62 and STUB1 to facilitate PDCoV N protein degradation (56). In the context of selective autophagy, PEDV N protein acts as a substrate that undergoes E3 ubiquitin ligase-mediated ubiquitination. Subsequently, they are targeted to lysosomes for degradation via cargo receptors (32, 36). During virus infection, host cells will inhibit the virus replication by regulating various intracellular signaling pathways. However, the antiviral function of ALDH1L1 has rarely been reported. In this study, we found that ALDH1L1, as one of the host antiviral proteins, would be upregulated to inhibit PEDV infection. Furthermore, we found that ALDH1L1 interacted with the E3 ubiquitin ligase STUB1 to ubiquitinate the PEDV N or E protein and recruited the cargo receptor TOLLIP to recognize and transport ubiquitinated N or E protein for degradation. This mechanism shares commonalities with the TRIM family-mediated antiviral selective autophagy pathway and the P62/SQSTM1-mediated antiviral selective autophagy pathway in terms of ubiquitination modification dependence, lysosomal degradation pathways, and viral inhibition effects. However, the antiviral mechanism mediated by ALDH1L1 shows significant differences in the composition of participating proteins, mode of action, and virus-targeting specificity. This suggests that host cells have evolved diverse and refined antiviral selective autophagy defense pathways in response to different viral infections.

## MATERIALS AND METHODS

### Cell culture and virus

Dulbecco’s modified Eagle’s medium (Sigma-Aldrich, D6429) supplemented with 10% fetal bovine serum (Gibco, 10099141) was used to culture human embryonic kidney HEK 293T cells (ATCC, CRL-11268) and African green monkey kidney Vero cells (ATCC, CCL-81). Modified Eagle’s medium (Gibco, 11095080) was used to maintain porcine kidney LLC-PK1 cells. These cells were generously supplied by Dr. Rui Luo (Huazhong Agricultural University, Wuhan, China). All cell lines were incubated in a 37°C humidified incubator with 5% CO₂. JS-2013 strain, a PEDV variant, was isolated and stored at our laboratory (32).

### Antibodies and reagents

The anti-ALDH1L1 antibody (A7707) used was obtained from ABclonal Technology. Anti-ACTB/β-actin antibody (66009-1-Ig), anti-GST antibody (HRP-66001), anti-STUB1 antibody (68407-1-Ig), anti-TOLLIP antibody (68170-1-Ig), horseradish peroxidase-labeled anti-rabbit and mouse antibodies, and IgG antibodies (SA00001-1, SA00001-2) were supplied by Proteintech. BafA1 (54645), MG132 (M7449), CQ (PHR1258), anti-FLAG M2 (F1804), and HA-tag (H6908) were purchased from Sigma‒Aldrich. Antibodies against the PEDV N protein were developed in our laboratory.

### Plasmids and transfection

Homologous recombination cloning was used to develop plasmid constructs by using a corresponding one-step kit (C112–02, Vazyme Biotech). Cell transfection was performed using Lipofectamine 3000 (L300015, Invitrogen) (80%–90% confluency). siRNAs were designed by GenePharma (Shanghai) and transfected into cells (50%–70% confluency) using Lipofectamine RNAiMAX (13778150, Invitrogen).

### RT-qPCR

Total RNA was isolated using either the FastPure Viral DNA/RNA Mini Kit (RC311) or the Cell/Tissue Total RNA Isolation Kit (RC112) (both Vazyme Biotech). Purified RNA was reverse transcribed into cDNA using the HiScript III RT SuperMix for qPCR (+gDNA wiper) (R323, Vazyme Biotech). The subsequent RT-qPCR process was conducted according to the protocols of the SYBR Premix Ex Taq Kit (q711, Vazyme Biotech).

### Western blotting

After rinsing the cells in cold phosphate-buffered saline, they were lysed for 15 min on ice using a protease inhibitor (SB-WB016, Share-bio) containing RIPA buffer (89901, Thermo Fisher Scientific). The resulting lysates were mixed with 5× SDS‒PAGE loading buffer (SB-PR037, Share-bio) for electrophoretic separation. Subsequently, proteins were transferred onto nitrocellulose membranes (10,600,001; GE Healthcare). The membranes were probed with specific primary and secondary antibodies. An enhanced chemiluminescence (ECL) reagent (SB-WB012, Share-bio) was used according to standard protocols to detect proteins.

### Co-IP assay

Cells were harvested in protease inhibitor-containing lysis buffer and incubated with anti-Flag Dynabeads (10,004D, Thermo Fisher Scientific). The Dynabeads were washed three times with 0.02% phosphate-buffered saline + Tween. Then, proteins were eluted from the beads using glycine buffer (pH 2.8). The resulting eluates were subjected to western blotting.

### GST affinity isolation assay

First, PEDV N, E, ALDH1L1, STUB1, and TOLLIP sequences were cloned. Then, they were inserted into Pcold-GST vectors. For the GST pull-down assays, recombinant proteins were expressed in *Escherichia coli* BL21 cells and purified according to the guidelines of a Protein Interaction Kit (21516, Thermo).

### Immunofluorescence assay

Cell samples were fixed with 4% paraformaldehyde (P6148) and permeabilized with 0.1% Triton X-100 (9284) (both Sigma–Aldrich). Thereafter, the cells were probed with 3% bovine serum albumin dilutions of designated primary and secondary antibodies for 60 min at 37°C. The stained cells were finally visualized under a confocal immunofluorescent microscope (Carl Zeiss, Germany).

### Statistical analysis

Intergroup comparisons were made using a two-sided Student’s *t*-test using Prism version 5 (GraphPad Software). *P*-values of <0.05, <0.01, and 0.001 were used to indicate statistical significance. All values are presented as the means of triplicate measurements.

## Data Availability

All data are contained within the article.
